# An Update on Prebiotics and on Their Health Effects

**DOI:** 10.3390/foods13030446

**Published:** 2024-01-30

**Authors:** Antonio Bevilacqua, Daniela Campaniello, Barbara Speranza, Angela Racioppo, Milena Sinigaglia, Maria Rosaria Corbo

**Affiliations:** Department of the Science of Agriculture, Food, Natural Resources and Engineering, University of Foggia, 71122 Foggia, Italy; antonio.bevilacqua@unifg.it (A.B.); daniela.campaniello@unifg.it (D.C.); barbara.speranza@unifg.it (B.S.); angela.racioppo@unifg.it (A.R.); milena.sinigaglia@unifg.it (M.S.)

**Keywords:** prebiotics, health, outcomes, human, pathologies

## Abstract

Prebiotic compounds were originally defined as “a nondigestible food ingredient that beneficially affects the host by selectively stimulating the growth and/or activity of one or a limited number of bacteria in the colon, and thus improves host health”; however, a significant modulation of the definition was carried out in the consensus panel of The International Scientific Association for Probiotics and Prebiotics (ISAPP), and the last definition states that “prebiotics are substrates that are selectively utilized by host microorganisms conferring a health benefit”. Health effects of prebiotics compounds attracted the interest of researchers, food companies and Regulatory Agencies, as inferred by the number of articles on Scopus for the keywords “prebiotic” and “health effects”, that is ca. 2000, for the period January 2021–January 2024. Therefore, the aim of this paper is to contribute to the debate on these topics by offering an overview of existing knowledge and advances in this field. A literature search was performed for the period 2012–2023 and after the selection of the most relevant items, the attention was focused on seven conditions for which at least 8–10 different studies were found, namely colorectal cancer, neurological or psychiatric conditions, intestinal diseases, obesity, diabetes, metabolic syndrome, and immune system disorders. In addition, the analysis of the most recent articles through the software VosViewer version 1.6.20 pointed out the existence of five clusters or macro-categories, namely: (i) pathologies; (ii) metabolic condvitions; (iii) structure and use in food; (iv) immunomodulation; (v) effect on gut microbiota.

## 1. Definition of Prebiotics and State of the Art

The concept of prebiotics was introduced in 1995 by Gibson and Roberfroid [1] as “a nondigestible food ingredient that beneficially affects the host by selectively stimulating the growth and/or activity of one or a limited number of bacteria in the colon, and thus improves host health”. Although revised several times, the main elements were retained over two decades.

Based on this definition, Roberfroid [2] highlighted the three main elements for prebiotic classification and pointed out as main criteria the resistance to mammalian enzymes in gastric environments, the fermentation by intestinal microbiota and the selective stimulation of some groups of bacteria; thus, he proposed the introduction of a prebiotic index, as the increase in bifidobacteria expressed as the absolute number of new cfu/g of feces divided by the daily dose (in grams) of prebiotic ingested.

Although widely diffused and cited (more than 10,000 times in January 2024), many researchers expressed some concerns about it, for example, the introduction of a new generation of compounds, different from fructans, which do not fit with this definition, or the emergence of new classes of probiotic microorganisms, etc. [3].

A revised definition was proposed by Bird et al. [4] and Bindels et al. [5]. Bird et al. [4] wrote that prebiotics are ‘undigested dietary carbohydrates’ that are fermented by colonic bacteria producing short-chain fatty acids (SCFA) as end products. In addition, Bindels et al. [5] proposed that selectivity and specificity were no longer relevant criteria.

The term prebiotic experienced a significant modification in the last consensus panel of The International Scientific Association for Probiotics and Prebiotics (ISAPP), and the current accepted definition states that “prebiotics are substrates that are selectively utilized by host microorganisms conferring a health benefit” [6].

This new definition does not take specifically into account the fermentation as the main metabolic route for prebiotic utilization; moreover, there are two main requisites, namely a selective utilization by host microorganisms, and the effect on microbiome [6].

Thanks to these amendments, there are several novel elements that should be considered:

(a) Although most current prebiotics are administered orally, they can also be administered directly to other microbially colonized body sites, such as the vaginal tract or the skin.
(b) The health benefits of prebiotics do not include only the modulation of several taxa in gut microbiota, but other positive effects are possible, including cardiometabolism (reduction in blood lipid levels, positive action on insulin resistance), hyperlipidemia, mental health (production of metabolites that influence brain function, energy, and cognition), bone (increased mineral bioavailability), direct and indirect effects on neurovegetative activity and antioxidant activity [6,7,8,9].
(c) Most prebiotics are carbohydrates/polysaccharides of vegetable origins (FOS, fructooligosaccharides; GOS, galactooligosaccharides; MOS, mannanoligosaccharide; XOS, xylooligosaccharide; generally, all with a polymerization degree between 4 and 30), but several other compounds fit with this new definition and can be considered as prebiotics, that is human milk oligosaccharides (HMO), phenols and other phytochemicals, conjugated linolenic acid (CLA), and PUFA (polyunsaturated fatty acids). An example of the possibility of including phenols in the class of prebiotic compounds could be found in Zhang et al. [9].
(d) Dietary fibers could be included in the prebiotic class if they are readily fermentable by host microbiota and cannot be used by host enzymes of the gut. Although some other compounds could fit the main requisites, they are not included among prebiotics (fat, proteins, less fermentable dietary fibers, vitamins).

The definition of 2017 is the main topic of an ongoing debate in the scientific community, but it could be considered as the basis to analyze the existing knowledge on prebiotics.

The link of prebiotics with the amelioration and/or the ability to counteract the side effects of several pathologies is a topic of relevant interest for consumers, Regulatory Agencies, and scientists. A comprehensive but not exhaustive view of research trends was carried out through a search on Scopus in January 2024 and gave ca. 2000 articles as an output for the period January 2021–January 2024 using the keywords “prebiotics” and “health effects” as inputs. This first search does not constitute the main literature analysis for the following sections, but a sort of introduction and state of the art.

The analysis of the keywords of all these 2000 articles through the software VosViewer version 1.6.20 pointed out the existence of 1000 items recurring at least five times. The analysis of references through bibliometric software could have possible bias, as clustering or the whole analysis could be affected by the algorithms used by the software itself. In the case of VosViewer, the main variable affecting the analysis is the organization of items into clusters depending on the times each item or keyword occurs, as well as the repetition of some combinations of keywords (that is if a term is always connected to the same words). Finally, another main requisite of VosViewer is the organization of clusters in descending order of items, that is the first cluster is that with more items, while the last cluster contains the lowest number of items. Despite these limitations, analysis through a bibliographic software constitutes the easiest way to gain an overview on a topic, without the need for reading all articles. 

**Figure 1:**
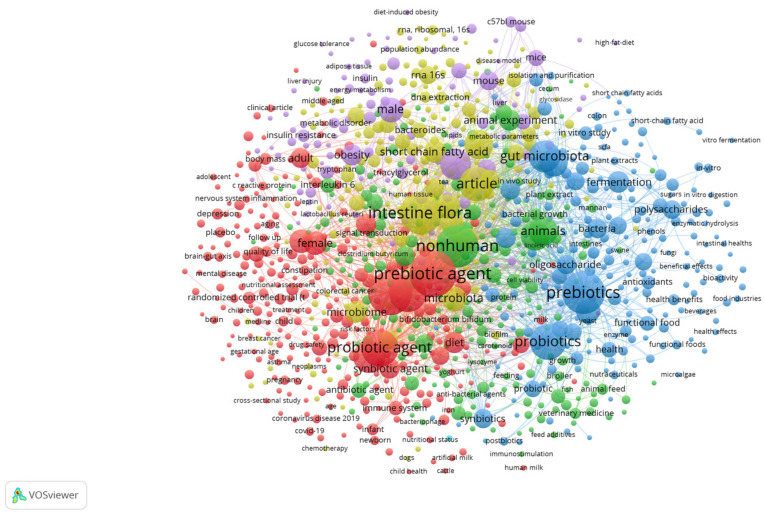
VosViewer analysis of the main items of “Prebiotics and Health Effects” on Scopus (November 2023). Red, combination of prebiotics and probiotics; Green, immunomodulation; Blue, use of prebiotics in food; Brown, effect on gut microbiota; Purple, metabolic conditions.

The items found on Scopus were organized by the software into five main clusters, each connected to a main topic or macro-category (Figure 1 and Table S1). The first cluster (red colour; 279 items) relies upon the effects of prebiotics, alone or combined with some probiotics (*Bifidobacterium bifidum*, *Bifidobacterium longum*, *Bifidobacterium infantis*, *Lactiplantibacillus plantarum*, *Lacticaseibacillus rhamnosus*, *Lactobacillus acidophilus*, among others), on neurological pathologies, as well as on the amelioration of chronic conditions, and respiratory diseases (for example COVID-19), in infants or adults.

A second cluster (green; 226 items) is connected to the effect of prebiotic on a general immunomodulation of the host, as evidenced by the main keywords of this group (antigen, immunity, interleukines, antibodies, Toll-like receptors, etc.). A third cluster (blue; 215 items) is connected to the use of prebiotics in foods, as well as on their molecular structure, and on the mode of delivery (normal components of food formula, active ingredients of capsules for a controlled release, etc.).

Cluster 4 (brown; 175 items) addresses the effects of prebiotics on gut microbiota, as well its modes of action (modulation of gut microbiota), with an increase in beneficial bacteria (*Akkermansia*, *Faecalibacterium*, bifidobacteria, lactobacilli), and a reduction in bacteria with side effects (clostridia), while the last important cluster (cluster 5, purple; 104 items) is connected to the effect of prebiotics on metabolic conditions (type 2 diabetes, metabolic syndrome, hyperglycemia, insulin resistance, fatty liver, fat amount in the body, etc.). Thus, the software reveals the interest of worldwide researchers for 5 macrocategories in prebiotic research, summarized by the following keywords: (i) pathologies; (ii) metabolic conditions; (iii) structure and use in food; (iv) immunomodulation; (v) effect on gut microbiota.

The interest in prebiotics is also summarized by the number of registered studies on clinicaltrials.gov (595 studies on 10 January 2024, among which 362 completed and 12 terminated, although results for only 16 studies were posted on the website). 

The main goal of this review is to contribute to the debate on the health effects of prebiotics, offering an update of existing knowledge, as well as focusing on the recent articles recovered in the literature, with a synopsis on the most interesting articles and their outputs.

## 2. Methodology for Literature Review

The literature review was carried out in December 2022, June 2023, and November 2023 on Scopus and PubMed; the following key words were used: prebiotics (or prebiotic compound), health effects, clinical studies, prebiotic and food, prebiotic output; the keywords were also combined, and the timeframe at least for a first search was from 2012 to 2022 (or 2023).

As a result, more than 3000 articles were found, and a first screening was carried out using some exclusion criteria:

(a) If, in an article, prebiotics had been combined with probiotics, the effect of prebiotics should be easily differentiated by those of probiotic microorganisms.
(b) Review articles were generally excluded, unless for definitions or to recover articles not found on PubMed or Scopus.
(c) If the compound tested did not fit with the main requisites of prebiotics, the article itself was excluded.
(d) Studies with only qualitative or not measurable outputs were excluded.

After this first screening, ca. 500 articles underwent to the second step by analyzing keywords and the abstracts, and by authors’ choice the exact definition of the pathological conditions, as well as experiments performed on animal models and in human volunteers and not only in laboratory conditions, were the main inclusion criteria. As a result of this second screening, ca. 140 articles were selected and used for literature review. As a final step, the articles were organized in 7 groups or pathological conditions, for which at least 8–10 different experiments/articles had been found, that is colorectal cancer, neurological or psychiatric diseases, intestinal diseases, obesity, diabetes, metabolic syndrome, immune system disorders.

The paper addresses the advances in knowledge on prebiotic effect for these pathologies.

## 3. Colorectal Cancer

Prebiotics could modify and positively affect the intestinal microbiota in patients affected by colorectal cancer (CRC) (Table S2A). Inulin alone and in combination with GOS increased the production of SCFA [10,11,12], which probably determined a reduction (49.9%) in the number of colon polyps [11]. 

COS (chitosan depolymerised oligomers) had a positive influence on CRC, through an increase of *Akkermansia* (butyrate-producing microorganism) and *Cladosporium* spp. and a reduction in *Escherichia*, *Shigella, Enterococcus,* or *Turicibacter* levels [13].

Ohara et al. [14] observed the synergistic effect between FOS and *B. longum* which led to an increase in SCFA content and a suppressive effect on *Bacteroides fragilis* enterotoxin (ETBF) and on putrefactive bacteria. 

In addition, marked anti-cancer properties were shown also by complex matrices with prebiotic action, such as Acacia gum with *Lpb. plantarum* [15], Yacon (known as the potato of diabetics) [16], seeds of Jabuticaba (an exotic fruit tree native to Brazil also known as grape tree) with *Lactobacillus delbrueckii* subsp. *bulgaricus* [17], jujube polysaccharides [18] and polysaccharides isolated from Nostoc commune Vaucher [19]. 

Finally, the combination of *Clostridium* spore-dextran plays an anti-tumor role in laboratory mice [20]. The spores of *Clostridium butyricum* were coated by dextran and orally administered; dextran fermentation by *Cl. butyricum* led to SCFA production, which in turn probably can inhibit growth and the tumor invasion of the CRC.

## 4. Psychological and Neurological Conditions

### 4.1. Cognitive Functions

Several researchers reported a possible effect of prebiotic compounds on stress and cognitive functions (Table S2B). Berding et al. [21] studied the effects of the consumption of vegetables, fermented foods, and prebiotics in adult subjects through Cohen’s scale and found a reduction in perceived stress, while Mysonhimer et al. [22] only found a positive reading of *Bifidobacterium* spp. after the consumption of FOS without a clear connection with mental health. 

Prebiotics could also affect cognitive functions. For example, Azuma et al. [23] studied the effect of a beverage containing inulin on Japanese women or men (50–80 years) and assessed biochemical and immunological parameters, the quali-quantitative composition of the microbiota of fecal samples, the cognitive functions through Cognitrax (a computer-based battery of cognitive function tests), and quality of life on eight scales (physical functioning, role physical, bodily pain, general health perceptions, vitality, social functioning, role emotional, and mental health); the results revealed the improvement in the scores of three domains of cognitive functions (attention, flexibility, and executive functions), probably linked to an increase in bifidobacteria and to a slight modulation of some inflammatory markers.

A possible effect on attention and on some other cognitive functions (including flexibility) was also found by Berding et al. [24], who studied the effect of polydextrose. These authors concluded that the improvement in the cognitive functions could be the result of the modulation of *Ruminococcus* 5, which in turn could be responsible for the decrease in some inflammatory markers.

### 4.2. Stress, Anxiety, and Depression

Prebiotics could also play a role on stress, anxiety, and depression (Table S2B), probably linked to a reduction in perceived stress [24], as a result of the modulation of *Bifidobacterium* spp. or of other taxa of gut microbiota [22]. 

Leo et al. [25] used α-lactalbumin (ALAC, a seroprotein with high biological value) combined with sodium butyrate (NaB), a postbiotic, to evaluate the effects on anxiety and depression on mice. This combination resulted in a valuable aid against depressive phenomena and anxious states by relieving symptoms and by reducing intestinal inflammation; in fact, the administration of both compounds resulted in behavioral improvements (improved sociability and memory and reduction in repetitive behavior) and increased motility [25]. According to the authors, ALAC would act on the intestinal composition and NaB would show a direct effect on the brain; moreover, NaB is a histone deacetylase inhibitor (hdaci) playing a role in neurodegenerative and neurological developmental diseases including epilepsy.

The role of prebiotics on depression is controversial, although preliminary data suggest the existence of possible correlation, as hypothesized by Tarutani et al. [26], who reported an improvement in the self-efficacy scores after the consumption of galactosylsucrose in patients with depressive episodes.

### 4.3. Autism

Table S2B reports the effects of prebiotics on ASD (autism spectrum disorders). B-GOS positively affected behavioral stereotyping, the levels of sociality, and lethargy and improved the qualitative composition of gut microbiota, increasing the concentration of bifidobacteria and other useful microorganisms [27,28,29].

Another effect resulting from the use of GOS combined with *Limosilactobacillus reuteri* and *B. longum* was a higher survival of probiotic strains, suggesting that GOS exerts a protective effect [27].

A restriction diet (free of gluten and casein, responsible for inflammation phenomena), associated with the intake of B-GOS, was administered to autistic children with positive effects on sociality and behavior. In addition, the prebiotic acted as a growth stimulator of *Faecalibacterium prausnitzii*, an anaerobic butyrate-producing microorganism in the human colon [30].

### 4.4. Schizophrenia and Parkinson

Prebiotics were also studied as active components in controlled trials on patients affected by schizophrenia and Parkinson. The data should be carefully confirmed and corroborated by other studies, due to the complexity of these pathologies and to the high number of variables playing a role, but there are some promising results, which suggest the possibility of using prebiotics as co-adjuvants to ameliorate the symptoms.

In particular, the consumption of raw materials with prebiotics (green leafy vegetables, high-fiber fruit, whole grains) improved the general cardio-metabolic profile in patients with schizophrenia spectrum disorders [31], while inulin, resistant starch, resistant maltodextrin, and rice bran played an active role in reducing the markers of inflammation (plasma zonulin and stool calprotectin), positively affected gut microbiota composition with an increase in SCFA, had a clinical impact leading to reduced severity of motor and non-motor Parkinson’s disease symptoms and improved gastrointestinal function [32].

## 5. Intestinal Diseases

### 5.1. Inflammatory Bowel Disease

About 25 years ago, Kennedy et al. [33] demonstrated the effectiveness of inulin in relieving inflammatory bowel disease (IBD) through a study conducted on mice with colitis provoked by dextran sodium sulfate (DSS); the daily oral administration of the prebiotic led to an increase in indigenous lactobacilli in the cecum and to a reduction in the pH of the colon. Moreover, the mucosal inflammation and histological damage scores were reduced as well as a lower degree of mucosal damage was observed [33]. Several years later, Koleva et al. [34] combined inulin with FOS to feed transgenic rats and observed a reduction in intestinal inflammation and increased levels of intestinal bifidobacteria and lactobacilli. They also found a decrease in mucosal proinflammatory cytokines (Table S2C). 

Similar effects were observed by using resveratrol, in mice with DSS-induced colitis [35]; in fact, increased levels of *Bifidobacterium* and *Lactobacillus* were observed, along with lower amounts of *E. coli* and Enterobacteriaceae.

Other human studies showed the ability of inulin and FOS in combination with *Bifidobacterium* to reduce inflammation and TNF (Tumor Necrosis Factor) and IL-1a (Interleukine-1a) [36]. 

Valcheva et al. [37] fed 25 ulcerative colitis (UC) patients with 7.5 or 15 g/day of fructans for 9 weeks. Patients in the high-dose group showed a significant increase in colon butyrate production and improvement of colitis. Moreover, inulin and FOS improved clinical symptoms and *Bifidobacterium* population even in patients with Crohn’s disease who were exposed to these prebiotics for four weeks [38]. 

Lindsay et al. [39] studied the effects of FOS in patients with Crohn’s disease: 15 g of FOS were administered for 3 weeks in 10 patients. FOS supplementation reduced the HBI score (HBI, index assessing the degree of disease activity), increased fecal *Bifidobacterium* concentrations, and increased the percentage of IL-10-positive dendritic cells (DCs).

### 5.2. Irritable Bowel Syndrome

GOS, oligosaccharides, inulin, and fructans are the main prebiotics often involved in ameliorating irritable bowel syndrome (IBS) symptoms, although the results are controversial [40,41]. Azpiroz et al. [42] described the influence of prebiotics on anxiety level of IBS individuals and concluded that FOS significantly reduced anxiety scores and increased fecal bifidobacteria. Wilson et al. [43] concluded that prebiotics did not lead to an improvement in the symptoms of the disease but rather favored the increase in bifidobacteria. However, when 44 patients received GOS as prebiotic, not only was an increase in the number of bifidobacteria observed, but also some symptoms, such as flatulence, abdominal pain, and discomfort resulted improved [44].

### 5.3. Enteric Syndrome

Prebiotics positively affect enteric syndrome, a severe congenital enteropathy, characterized by intractable diarrhea in the first month of life, associated with growth retardation, facial dysmorphism, hair abnormalities and, in some cases, immune system disorders and intrauterine growth restriction [45]. It could be treated with antibiotics, but as reported by Ayala-Monter et al. [46], their improper use can cause bacterial resistance; thus, prebiotics and probiotics appear to be valid alternatives.

Supplementary Table S2C reports four studies carried out on animals (mice and weaned lambs), using GOS, exopolysaccharides (EPS), inulin [46,47,48], and catechins (substances with a prebiotic action known for their strong antioxidant properties) [45]. 

Each prebiotic compound can stimulate the growth of lactobacilli and bifidobacteria in the gut. For inulin, significant increase in the percentage of basophils, improvement in the body’s immune response, and significant reduction in diarrheal phenomena were also observed, while catechins showed a marked ability to stimulate SCFA production [45,48]. A synbiotic action of inulin *+ Lcb. casei*, compared to the sample treated only with inulin, favored the increase in lactobacilli and the reduction in total coliforms, improving the use of nutrients introduced with the diet [46].

## 6. Obesity

Supplementary Table S2D shows some articles on obesity and overweight. For example, COS, FOS and GOS determined an improvement in the gut microbiota dysbiosis with a marked anti-inflammatory activity, probably linked to SCFA production [49,50].

A common effect of flavanols, decaffeinated green and black tea polyphenols, aqueous extracts of tea, marc, cinnamon, inulin, vanillin, and lignans is the reduction in the Firmicutes/Bacteroidetes ratio [51,52,53,54,55,56]. This ratio is considered as a possible hallmark for obesity, as it is high in obese people and tends to decrease following weight loss. In fact, Magne et al. [57] observed the increased abundances of Firmicutes in obese animals and humans, due to the fact that they are more efficient in extracting energy from food than Bacteroidetes, thus promoting a higher calorie absorption and a consequent weight gain. However, in the case of following a low-calorie diet for 12 months, Bacteroidetes increased, with the consequent normalization of the Firmicutes/Bacteroidetes ratio, along with weight loss [57]. *Bacteroides* can reduce serum triglyceride levels, improve glucose intolerance, and counteract body weight gain [51].

Flavanols and aqueous extract of tea were also able to promote the growth of *A. muciniphila* [51,53] and similarly did other potential prebiotic compounds, such as cranberry extract, apple procyanidins, aqueous tea extracts, resveratrol, pterostilbene, and catechins [6,58,59,60,61].

The role of inulin-type fructans (ITF) (carbohydrates consisting of β-(2-1)fructosyl-fructose units) is also important, as they can modulate the intestinal microbiota composition in obese women by stimulating the growth of *F. prausnitzii* [62].

ITF, resveratrol, catechins, flavanols, promote the growth of bifidobacteria, which play an essential role in fighting obesity [6,28,59,62] as they modulate the secretion of ghrelin, a hormone that regulates the sense of appetite in vitro, highlighting their therapeutic potential [27]. 

A positive effect on *Bifidobacterium* spp., also linked to a modulation of fecal calprotectin and to an increase in rumenic and linolenic acids, was evidenced by Neyrinck et al. [63] during a 3-month, multicentric, single-blind, placebo-controlled trial. The most important outcome of this study was the strong reduction in calprotectin, thus emphasizing the potential interest of prebiotic intake to combat gut inflammatory disorders occurring with obesity.

This effect on inflammation was also reported by Crovesy et al. [64], who combined FOS with a probiotic (*B. animalis* subsp. *lactis*), and by Lyon et al. [65], who studied the effect of a combination of inulin from chicory with a complex mixture of probiotic microorganisms (lactobacilli, bifidobacteria, *Bacillus*, *Streptococcus*, *Saccharomyces*). 

Other compounds (soy isoflavones, pomegranate extract, arctic berries, pollen extract, and genistein) positively affected gut microbiota composition and favored weight loss [66,67,68,69,70,71].

Positive effects of prebiotics on obese patients also include a reduction in the levels of cortisol with a direct effect on sleep quality [72], a significant decrease in plasma triglycerides [73], and a reduction in waist and hip circumferences [70].

## 7. Diabetes

COS and ITF were the most used prebiotics in diabetes (Table S2E); these compounds, alone or combined with probiotics, exert various beneficial effects. Some studies on mice highlight that COS reduces hyperglycemia and hyperlipidemia and prevents obesity. In addition, it positively affects the composition of the gut microbiota; in fact, it favors the abundance of Firmicutes, Bacteroidetes and Proteobacteria [74], as well as Actinobacteria and Lachnospiraceae populations [75]. In addition, COS reduces blood glucose levels (BGLs) [75].

Just like COS, ITF also promotes a reduction in BGL; other effects are a reduction in fasting blood glucose (FBG), a lower Firmicutes/Bacteroidetes ratio, and increased levels of *Phascolarctobacterium*, *Lachnoclostridium* [76], *F. prausnitzii* and bifidobacteria [77]. 

In particular, Birkeland et al. [77] found that ITFs are responsible for the production of acetic and propionic acid; in fact, patients with diabetes have lower levels of butyrate-producing intestinal microorganisms and often occurs that the severity of the disease intensifies.

Zhang et al. [78] evaluated the interactions between plant extracts (bitter gourd extract, BGE and mulberry leaf extract, MLE) and potential probiotics (*Lcb. casei* K11 and *Lacticaseibacillus paracasei* J5) on mice; both extracts provided interesting results. In fact, microbial targets showed a marked vitality in the gastrointestinal tract. In addition, the interactions *Lcb. casei* K11-BGE and *Lcb. casei* K11-MLE significantly reduced BGL and improved insulin resistance in diabetic mice. *Lcb. casei* K11 with both plant extracts also modulated lipid metabolism, proinflammatory cytokine levels and oxidative stress; in addition, it led to an improvement of glucagon-like peptide-1 (GLP-1) secretion, SCFA levels, and free fatty acid receptor 2 (FFAR2) upregulation.

Diabetes was also chosen as the target for other prebiotics, like dextran or commercial formulas. Resistant dextran and maltodextrin were tested by Saleh-Ghadimi et al. [79] in a randomized controlled trial on female obese type 2 diabetic patients; sleep quality and quality of life were assessed by the Pittsburgh Sleep Quality Index and SF-36 health survey, respectively, along with serum bacterial endotoxin, fasting blood sugar, glycosylated hemoglobin, pro-inflammatory/anti-inflammatory biomarkers, and biomarkers of hypothalamic–pituitary–adrenal axis function. The results suggested an improvement of the quality of life of patients, probably linked to the modulation of some physiological parameters (glycemia, metabolic endotoxemia and inflammatory cytokines).

In a recent study [80], a commercial formula, composed of inulin and glucans, was tested on young patients affected by type 2 diabetes and the main effect was a modulation of gut microbiota after 1 week and 1 month, while stool frequency and gastro-intestinal symptoms were not affected.

## 8. Metabolic Syndrome

N acetyl-chitooligosaccharide (NACOS) and proanthocyanidins extracted from grape seeds resulted in a reduction in Firmicutes [81,82,83]; however, most studies with phenolic extracts did not produce definitive clinical evidence, as patients generally involved in the trial are poly-medicated subjects affected by several variables [84] (Table S2E). 

NACOS improved glucose tolerance and inhibited lipid accumulation in the liver [82]. In addition, by monitoring fasting blood glucose (FBG), mice fed with NACOS actually had lower fasting glucose, and by measuring plasma insulin, it was found that feeding NACOS greatly promoted insulin secretion [81].

Concerning pro-anthocyanidins of grape seeds, they exerted a positive effect on satiety-related enterohormones (glucagon-like-peptide-1, GLP-1; ghrelin) as they led to a significant increase in GLP-1, and, therefore, to an improvement in glucose tolerance, and an induction of satiety, strengthened by the increase in ghrelin [83].

## 9. Osteoporosis

FOS and GOS were essential to a better absorption of calcium, better density, and resistance to bone wear [85,86,87,88] (Table S2F).

In a study conducted on animal models, the treatment with FOS recorded higher levels of serum alkaline phosphatase (ALP a marker enzyme of bone formation, used in the diagnosis of skeletal and liver diseases) and femurs with higher resistance. Increased bone density can lead to greater bone strength, reducing the risk of fracture [85].

Interesting was the study conducted by Johnson et al. [87] who compared antibiotics and prebiotics administered in mice. After 10 weeks of treatment with alendronate (a drug given for osteoporosis, especially in menopausal women), bone mineral density increased by 7.31%. The best results were obtained for FOS + dried prune treatment, which led to an increase of 36%. Hence, the combination of these two compounds has shown results that are equivalent to and can surpass those of conventional drugs [82]. Other data were reported by Wu et al. [89], who studied calcium absorption in premenopausal women with history of RYGB (Roux-en-Y gastric bypass). The trial was based on soluble corn fiber, and the results suggested a possible effect due to a shift in microbiota composition. A partial effect with soluble corn fiber was also found after 6 months in Malaysian preadolescent with an increase in bone density, but not after 1 year [90].

Therefore, to improve the symptoms related to osteoporosis, the use of prebiotics can represent a valid alternative to conventional drugs, which, in addition to being particularly expensive, can also have various side effects.

## 10. Immunosenescence

Several articles focused on immunosenescence; it consists of the gradual deterioration of the immune system, due to natural age advancement; it involves both the host’s capacity to respond to infections and the development of long-term immune memory. 

In immunosenescence, gut microbiota composition is not constant but change with aging, and these changes have been linked to declines in immunity; however, it has been demonstrated that the maintenance of a “youthful” and “healthy” gut microbiota could positively affect by delaying immunosenescence [91]. Therefore, probiotics and prebiotics perform the function of reducing the proinflammatory response and improving innate immune dysfunction in the elderly.

Syringaresinol (SYR), a lignan occurring in plant foods (oilseeds, cereal brans, and various berry seeds) act as antioxidant, antistress, antitumorigenic, and anti-inflammatory compound; although at present the mechanism is not yet well understood, the compound can delay immunosenescence by modulating the immune system and the composition of gut microbiota. Si-Young et al. [91] reported that SYR effectively delayed immunosenescence by increasing the number of total T lymphocytes, which identify the antigen and activate the immune response, by implementing a protection against infections by intracellular microorganisms such as viruses and some bacteria [91]. Moreover, SYR reduced the Firmicutes/Bacteroidetes ratio; furthermore, it markedly increased the *Bifidobacteriium* and *Lactobacillus* (*B. animalis*, *Lactobacillus johnsonii*, *Lim. reuteri*) population, compared to control samples. Conversely, potentially opportunistic genus members, Bacteroidaceae, *Bacteroides vulgatus* and *Staphylococcus lentus*, were adversely affected [91].

A positive effect on microbial population was also observed in GOS, B-GOS, FOS, chicory inulin treatments towards humans [92,93,94]; these compounds support the growth of *Bifidobacterium* and *Lactobacillus* spp., ensuring a better state of intestinal health. B-GOS, GOS, and FOS stimulate the production of SCFA, counteracting inflammatory states [92,94].

## 11. Conclusions

It is well known that prebiotics could exert a significant impact on human health, through direct and indirect mechanisms, but the modulation of gut microbiota remains the key focus for most positive outcomes of clinical studies.

However, the data and evidence collected in this review suggest the possibility of using prebiotics in a wide variety of conditions, with a many possible outcomes, including the amelioration of the symptoms in several pathological conditions (autism, CRC, IBD, osteoporosis, etc.) or the improvement of the quality of life, through the positive action on some cognitive functions as well as the reduction in inflammatory and pro-inflammatory conditions.

The results hereby collected, however, do not provide robust evidence on the exact mechanisms and on possible pathways, but suggest possibilities or hypotheses.

It is worth mentioning that the core of prebiotic action is the shift of gut microbiota towards eubiosis, linked to the production of increased levels of SCFA and a taming effect on inflammatory conditions; these actions are probably responsible for the clinical outcomes (or secondary effects), but how primary effects and secondary outcomes are linked is still not clear.

Some critical points should be addressed for a more robust focus on the actual effects of prebiotics, that is, the use of standardized protocols, in terms of compounds (uniformity in chemical structure for some classes, as well similar degree of polymerization), doses, modes of drugging (with foods or as beads), duration of the clinical trials, and kind of supplementation (alone or with probiotic microorganisms). All these variables are confounding factors, able to strongly influence the outcome.

“Tell me what you eat and I shall tell you who you are”: this famous sentence by Anthelme Brillat-Savarin can be also applied to prebiotics, as they are the main ingredients of many foods and can support physical and mental health and well-being; this evidence is strengthened by science. However, now it is important to translate scientific achievements in guidelines and eating habits, spreading the knowledge and the advances of research in consumers’ awareness.

## Data Availability

The original contributions presented in the study are included in the Supplementary Materials; further inquiries can be directed to the corresponding author.

## Supplementary Materials

The following supporting information can be downloaded at: https://www.mdpi.com/article/10.3390/foods13030446/s1, Table S1: Items of VosViewer clusters; Table S2: Health effects of prebiotics; (A) Colorectal Cancer; (B) Neurovegetative Diseases and Cognitive Functions; (C) Intestinal Diseases; (D) Obesity; (E) Type-2 Diabetes and Metabolic Syndrome; (F) Osteoporosis and Immunity. References [95,96,97,98] are cited in the Supplementary Materials.
