# Supplementary material for: An Update on Prebiotics and on Their Health Effects
Source: Foods. 2024 Jan 30;13(3):446. doi: 10.3390/foods13030446 (PMC10855651; doi:10.3390/foods13030446)
Supplement: Supplementary file 1 [file foods-13-00446-s001.zip › supplementary table S2.pdf]

Table S2A: Effects of prebiotics in Colorectal Cancer

**Prebiotic**	**Effects**	**Model**	**Reference**
Inulin and GOS	In situ production of SCFA<br>Significant increases of *Bifidobacterium* spp.	Animal	10
GOS	Significant increase in beneficial populations, such as *Bifidobacterium* spp.; 56.9% increase in propionate production<br>Increase of Bacteroidetes and reduction of Firmicutes populations<br>Reduction of the genus *Desulfovibrio* spp.<br>Increase in the genus *Phascolarctobacterium*	Animal	12
Inulin	Increase in situ production of SCFA (propionic acid and butyric acid)<br>Increase of Bacteroidetes and Prevotellaceae<br>Reduction of the Firmicutes<br>Increase of the genus *Blautia*<br>Increase in fecal mass<br>Reduction (49.9%) in the number of colon polyps	Animal	11
COS	Reduction of *Escherichia* spp., *Shigella* spp., *Enterococcus* spp. and *Turicibacter*<br>Increase of *Akkermansia* spp., butyrate producing bacteria and *Cladosporium* spp.	Animal	13
FOS (25%)<br>XOS (25%)<br>polydextrose (25%)<br>Resistant dextrin (25%)	Improvement of serum immunological indicators<br>Significant increase in levels of igG and igM, igG, igA, total B lymphocytes and T cells<br>Increased level of transferrin<br>Reduction of Bacteroidetes<br>Increase of *Bifidobacterium* spp. and *Enterococcus* spp.<br>Increase of harmless strains of *Escherichia* specie	Human	53
FOS + *Bifidobacterium longum*	Increased of SCFA in feces<br>Suppression of rotting bacteria in feces<br>Suppression of *Bacteroides fragilis* enterotoxin	Human	14
Acacia gum + *Lactiplantibacillus plantarum*	Reduction of TNF-α levels due to the anti-inflammatory activity of acacia gum<br>Reduction of β-glucuronidase levels	Animal	15

Yacon	Improved intestinal permeability<br>Increased production of epithelial mucus<br>Increased antioxidant activity<br>Reduction of TNF<br>Increased SCFA levels<br>Increased production of antibacterial defensins and anti-inflammatory cytokines, mainly IL-10	Animal	16
Jabuticaba seeds + *Lactobacillus delbrueckii* subsp.*bulgaricus*	Increase of Bacteroidetes<br>Reduction of Firmicutes<br>Reduction of β-glucoronidase, β-glucosidase, galactosidase, mucinase and nitroreductase (bacterial metabolizing enzymes), and consequent reduction in the incidence of colon cancer<br>Growth inhibition of *Bacteroides*, *Clostridium* and *Propionibacterium* spp.<br>Increase of *Lactobacillus* and *Bifidobacterium* spp.	Animal	17
JP	Significant decrease in Firmicutes/Bacteroidetes ratio<br>Strong activity in the regulation of dysbiosis and maintenance of microbial balance<br>Increased SCFA production	Animal	18
NVPS	Reduction in the number and size of tumors<br>Reduction in the incidence of CRC<br>Reduced Firmicutes/Bacteroidetes ratio<br>Increase in SCFA producing genera, including butyric producers (*Butyricicoccus*, *Butyrivibrio* and *Butyricimonas*) and acetic acid (*Lachnospiraceae* UCG 001, *Lachnospiraceae* UCG 006 and *Blautia*)	Animal	19
Dextran	Production of SCFA with an anti-inflammatory action	Animal	20

Table S2B: Effects of prebiotics in neurovegetative diseases and cognitive functions

**Prebiotic**	**Effects**	**Model**	**Reference**
	**Cognitive Functions**		
Inulin + *Bifidobacterium animalis* subsp. *lactis*	Improvements in scores of the neurocognitive index domain (overall cognitive function, overall attention, cognitive flexibility, and executive functions)<br>Reduction of some inflammation markers	Human	23
Polydextrose	Better performances in sustained attention<br>Increased abundance of *Ruminoclostridium* 5	Human	24
	**Stress**		
Prebiotic fruits and vegetables, fermented foods	Improvement of perceived stress in adults	Human	21
FOS and GOS	Increase of *Bifidobacterium* spp.<br>No clear effect on the markers of stress and inflammation	Human	22
	**Anxiety and Depression**		
ALAC+ sodium butyrate	Relieve the symptoms of anxiety<br>Reduction of intestinal inflammation<br>Reduction of the immobility time<br>Increased preference of sucrose, thereby reducing anhedonia.	Animal	25
Galactosylsucrose	Improvement of self-efficacy for patients with depression	Human	26
	**Autism**		
GOS + *Limosilactobacillus reuteri* and *Bifidobacterium longum*	Increased survival of probiotics<br>Increased levels of Firmicutes, *Akkermansia*, *Blautia* and *Actinobacter*<br>Reduction of Bacteroides and Verrucomicrobia<br>Increased concentrations of SCFA<br>Reduction of constipation, diarrhea and pain<br>Improvement in terms of lethargy, irritability and stereotypes	In vitro fermentation model: SHIME (Simulator of Human Intestinal	27

		Microbial Ecosystem)	
B-GOS	Increased amounts of creatinine, creatine, dimethyl-cine, dimethylanine, carnitine, citrate, adipate and trimethylamine-N-oxide in children's urine with ASD<br>Reduction of amino acid and lactate levels<br>Improvement of abdominal pain	Human	28
B-GOS	Significant increase of bifidobacteria, *Rosburia*, *Bacteroides*, *Atopobium*, *Faecalibacterium prausnitzii*, *Sutterella* spp.; rectal *Eubacterium*<br>Reduction of Veillonellaceae<br>Increased of acetate and lactate	Human	29
B-GOS combined with a casein and gluten free diet	Improvement in sociality and behavioural traits<br>Increased of *Faecalibacterium prausnitzii* and *Bacteroides* spp.	Human	30
ALAC+ sodium butyrate	Improvement of sociability and memory<br>Reduction of intestinal inflammation<br>Increased mobility<br>Reduction of repetitive behavior	Animal	25
Inulin + oligofructose	Improvement of intestinal permeability<br>Improvement of the behavioral field (reduction of repetitive behaviors)	Animal	95
**Schizophrenia**			
Green leafy vegetables, high-fibre fruit, whole grains	Improvement of cardio-metabolic profile in schizophrenia spectrum disorders	Human	31
**Parkinson**			
Inulin, resistant starch, resistant maltodextrin, rice bran	Beneficial biological changes in the microbiota, SCFA, inflammation, and neurofilament light chain<br>Reduction of the markers of inflammation (plasma zonulin and stool calprotectin)	Human	32

Table S2C: Effects of prebiotics in intestinal diseases

Prebiotic	Effects	Model	Reference
**Inflammatory Bowel Disease**			
Inulin	Increased level of indigenous lactobacilli;<br>Reduction of the pH of the colon;<br>Reduction of the mucosal inflammation and lower scores of histological damage	Animal	33
Inulin + FOS	Reduction in intestinal inflammation<br>Increased levels of intestinal bifidobacteria and lactobacilli;<br>Decrease in mucosal proinflammatory cytokines	Animal	34
Resveratrol	Increased levels of *Bifidobacterium* and *Lactobacillus*<br>Lower amounts of *E. coli* and Enterobacteriaceae	Animal	35
Inulin + FOS + *Bifidobacterium*	Reduction in intestinal inflammation and in the intestinal TNF and IL-1a levels	Human	36
fructans of inulin type	Increase in colon butyrate production and improvement of colitis	Human	37
Inulin + FOS	Improved clinical symptoms and increased level of *Bifidobacterium*	Human	38
FOS	Reduction of the HBI score<br>Increased fecal *Bifidobacterium* concentrations<br>Increase of the percentage of IL-10–positive DCs.	Human	39
**Irritable Bowel Disease**			
scFOS	Reduction of anxiety scores<br>Increase of Bifidobacteria population	Human	42
GOS	Increase in the number of Bifidobacteria<br>relief of flatulence, abdominal pain, and discomfort symptoms	Human	44
**Enteric Syndrome**			

GOS + Bifidobacteriaceae	Intestinal increase of bifidobacterial and lactic acid bacteria<br>Improved renal function	Animal	47
EPS + *Leuconostoc mesenteroides*	Growth stimulation of bifidobacteria, *Lactobacillus* spp. and SCFA<br>Increase in antioxidant activity<br>Stimulation of antioxidant activity<br>Improvement of skin immunity	Animal	48
Inulin + *Lacticaseibacillus casei*	Increase in basophils and lactobacilli concentration<br>Reduction of total coliform concentration in faeces<br>Reduction of diarrheal phenomena<br>Increased consistency of the faeces<br>Reduction of serum cholesterol concentration	Animal	46
Catechins	Increase of *Lactobacillus* spp., *Bifidobacterium* spp., *Akkermansia* spp., *Roseburia* spp. and *Faecalibacterium* spp.<br>Increased production of SCFA	Animal	45

Table S2D: Effects of prebiotics in Obesity

**Prebiotic**	**Effects**	**Model**	**Reference**
COS	Increased level of acetic acid, with reduction of propionic and butyric acid; *Bacteroides* increase in microbiota and reduction of Proteobacteria and Actinobacteria	Animal	49
FOS/GOS	Improvement of the microbiota dysbiosis<br>Increased inflammatory cytokines	Animal	50
Flavanols: epi-gallocatechin, epigallocatechin gallate, epicatechin, epicatechin gallate	Increase of *Akkermansia*, *Roseburia* and *Bifidobacterium*<br>Increased production of butyrate<br>Improvement of intestinal barrier function<br>Reduction of the Firmicutes/Bacteroidetes ratio<br>Reduction of translocation of endotoxins	Animal	51
Decaffeinated Green and Black Tea (GT and BT) polyphenols	Inhibition of weight gain in mice fed a high-fat diet<br>Firmicutes/Bacteroidetes ratio reduction<br>α-amylase and α-glucosidase inhibition in saliva and small intestine<br>Increased SCFA (acetate and propionate)	Animal	52
Aqueous extracts of tea	*Firmicutes* increase and decrease of Bacteroidetes<br>Potential growth of *Akkermansia muciniphila*	Animal	53
Marc and cinnamon extracts	Improvement of blood sugar<br>Reduction of fat mass increase<br>Enrichment in Firmicutes<br>Decrease in *Bacteroides*<br>Reduction of hepatic steatosis	Animal	54
Inulin	Increase of actinobacteria<br>Reduction of Clostridia<br>Reduction of girmicutes (*B. obeum*, *E. ruminantium*, *B. obeum*, *B. luti*, *B. faecis*, *R. faecis*, *Oscillibacter* spp.)<br>Growth stimulus of *Bacteroides* spp. (*B. uniformis*, *B. xilanisolveni*)	Animal	55
Vanillin and lignans	Reduction of Firmicutes<br>Increase of Bacteroidetes and Verrucomicrobia<br>Inhibition of lipopolysaccharide producing bacteria (LPS)<br>Improved sensitivity to insulin	Animal	56

	Stimulation of SCFA production		
Cranberry extract	Reduction of intestinal triglyceride content<br>Relief of intestinal inflammation and oxidative stress<br>Increase of *Akkermansia* spp.<br>Reduction of the weight gain	Animal	58
Apple Procyanidins	Increase of *Akkermansia muciniphila*<br>Improved sensitivity to insulin<br>Improvement in lipid catabolism	Animal	59
Resveratrol	Increase of *Lactobacillus* spp., *Akkermansia muciniphila* and *Bifidobacterium* spp.<br>Improved sensitivity to insulin	Animal	60
Pterostilbene	Increase of *Akkermansia muciniphila*<br>Improved sensitivity to insulin<br>Increased production of butyrate and propionate	Animal	61
Catechins	Increase of *Roseburia* spp., *Lactobacillus* spp., *Bifidobacterium* spp. *Akkermansia* spp. *Faecalibacterium* spp.	Animal	45
Inulin-type fructans (ITF)	Growth stimulation of Bifidobacteria and *Faecalibacterium prausnitzii*<br>Reduction of *Bacteroides intestinalis* and *Bacteroides vulgatus*	Human	62
Soy isoflavones	Increase of *Faecalibacterium*<br>Reduction in blood lipid levels<br>Improved immune function and intestinal permeability<br>Attenuation of oxidative damage<br>Increased fraction of beneficial bacteria producing SCFA (*Bacteroidetes* and Proteobacteria)	Animal	66
Pomegranate extract	Increase of *Bacteroides*, *Faecalibacterium*, *Butyricicoccus*, *Odoribacter* and *Butyricimonas*<br>Reduction of *Parvimonas*, *Methanobrevibacter* and *Methanosphaera*	Animal	67
Pomegranate extract + syringaresinol	Reduction of serum LBP, therefore polyphenols could modulate metabolic endotoxaemia	Animal	68
Arctic berries	Relief of intestinal infiammation induced by a high-fat diet<br>Reduction of hepatic steatosis<br>Prevention of hyperinsulinemia	Animal	58

Pollen extract	Reduction of lipid accumulation in serum and liver<br>Reduction of oxidative damage and inflammation<br>Increase of *Bifidobacterium* spp.	Animal	70
Genistein	Reduction of weight gain<br>Reduction of serum triglycerides<br>Improvement of the glucose tolerance	Animal	71
Chicory inulin combined with a probiotic mixture (20 billion CFU; lactobacilli, bifidobacteria, *Bacillus, Streptococcus, Saccharomyces*)	Reduction of BMI, body weight, and waist and hip circumferences<br>Reduced subclinical inflammation related to immunity	Human	65
Inulin-type fructan	Increase in *Bifidobacterium* content<br>Reduction of fecal calprotectin<br>Increase of rumenic and conjugated linolenic acid	Human	63
Galacto-mannans combined with *Bacillus coagulans*	Higher weight loss in patient of bariatric surgery<br>Significant decrease of plasma triglycerides	Human	73
Bifidobacterium animalis subsp. *lactis* and FOS	Changes in serum metabolite profile related to a decrease in inflammation	Human	64
FOS, yeast β-glucans, silymarin extract	Reduction of cortisol with effect on sleep quality	Human	72
Resistant dextrin	Modification of satiety and glycaemic responses	Human	96

Table S2E: Effects of prebiotics in type-2 Diabetes and Metabolic Syndrome

**Prebiotic**	**Effects**	**Model**	**Reference**
**Diabetes**			
COS	Reduction of hyperglycemia, hyperlipidemia<br>Prevention of obesity<br>Histological improvement in the liver of T2DM mice<br>Increased of Firmicutes, Bacteroidetes and Proteobacteria	Animal	74
COS	Reduction of BGL in diabetic mice;<br>Increase of Actinobacteria, Lachnospiraceae and Corynebacteriaceae populations	Animal	75
ITF + *Lactobacillus* spp.	Reduction of BGL<br>Reduction of FBG<br>Reduction of the Firmicutes/Bacteroidetes ratio<br>Increased of *Lactobacillus* spp., *Bacteroides*, *Phascolarctobacterium* and *Lachnoclostridium*	Animal	76
ITF	Increase in the intestinal level of Bifidobacteria<br>Increased concentration of acetic and propionic acids<br>Decrease of *Firmicutes*<br>Slight increase of *F. prausnitzii*	Human	77
Resistant Dextrin and maltodextrin	Resistant dextrin improves sleep and QOL in obese women with type 2 diabetes<br>Modulation of glycemia, metabolic endotoxemia and subsequently a decrease in biomarkers of inflammation and HPA axis activity	Human	79
Inulin-type fructans	Improvement incretin responses or glucose regulations	Human	97
Commercial prebiotic formula	Modulation of gut microbiota		80
**Metabolic Syndrome**			
NACOS	Promotion of insulin synthesis<br>Improved glucose tolerance	Animal	81

NACOS	Inhibition of lipid accumulation in the liver<br>Reduction of the Firmicutes/Bacteroidetes ratio<br>Reduction of *Desulfovibrio* spp.<br>Reduction of fasting blood sugar<br>Increase of the *Lactobacillus*/*Enterococcus* ratio		82
Pro-anthocyanidins from grape seeds	Reduction of cecal butyrate<br>Increase of *Bacteroides* and Proteobacteria<br>Decrease in Firmicutes<br>Significant increase of *Akkermansia muciniphila*	Animal	83
Pomegranate extract	Modulation of gut microbiota, depending also on the pharmacological therapy<br>Reduction of lipopolysaccharide-binding protein	Human	84
Inulin, inulin+traditional Chinese medicine (TCM), or inulin+metformin	Inulin alone or combined with metformin or TCM altered specific gut microbiota taxa but not the general diversity<br>Correlation of microbiota with lipids, uric acid, and glucose, with possible effects on MetS	Human	98

Table S2F: Effects of prebiotics in Osteoporosis and Immunity

**Prebiotic**	**Effects**	**Model**	**Reference**
**Osteoporosis**			
FOS	Increase in bone mass<br>Increase of butyrate concentration in the colon	Animal	85
FOS/GOS	Improved calcium, phosphorus and magnesium absorption<br>Reduction of bone loss in ovariectomy-induced rats	Animal	86
FOS and dried prunes	Improvement of bone mineral density<br>Improvement in bone recovery	Animal	87
FOS	Increased *Lactobacillus* and *Bacteroides* population<br>Increased calcium content in the femoral bone<br>Decrease of the level of urinary deoxypyridinoline and serum high-sensitivity C-reactive proteins.	Animal	88
Soluble corn fiber	Variable impact on calcium absorption in pre-menopausal women due probably to gut microbiota alteration	Human	89
Soluble corn fiber, with and without calcium supplementation	Increase bone density after 6 months in preadolescent	Human	90
**Immunity**			
Syringaresinol	Reduction of the Firmicutes/Bacteroidetes ratio<br>Increase of *Lactobacillus* and *Bifidobacterium* spp.<br>Reduction of *Akkermansia* spp.;<br>Reduction of serum level of LPS binding protein<br>Improvement of humoral immunity	Animal	91
B-GOS	Significant increase in *Bifidobacteria, Lactobacillus* spp., *C. coccoide, Enterococcus* spp.<br>Significant increases in phagocytosis and production of anti-inflammatory cytokine interleukin-10 (IL-10)<br>Significant reduction in the production of proinflammatory cytokines (IL-6, IL-1beta and tumor necrosis factor alpha)<br>Reduction of *Desulfovibrio* spp.	Human	92

Chicory inulin	Increase in the *Bifidobacterium* spp.	Human	93
GOS and FOS	Increased in bifidobacteria and butyrate	Human	94

List of abbreviations

1. **ADHD** = Attention Deficit Hyperactivity Disorder;
2. **ADOS-CSS** (Autism Diagnostic Observation Schedule – Calibrated Severity Score) = standard assessment tool in autism research;
3. **AN** = Anorexia Nervosa;
4. **ATEC** = Autism Treatment Evaluation Checklist;
5. **BDI** (Beck Depression Inventory) = depression assessment tool;
6. **BDNF** = brain-derived neurotrophic factor;
7. **BGL** = blood glucose levels;
8. **BMI** = Body Mass Index;
9. **CD** = Crohn's disease;
10. **CRP** = C-reactive protein;
11. **DCA** = secondary bile acid deoxycholic acid;
12. **DCs** = dendritic cells;
13. **FBG** = fasting blood glucose;
14. **FOS** = fructo-oligosaccharides;
15. **FPG** = fasting plasma glucose;
16. **FST** (Forced Swim Test) = rodent depression assessment test;
17. **GABA** = gamma-aminobutyric acid;
18. **HDL** = High Density Lipoprotein;
19. **IBS**= Irritable Bowel Disease;
20. **IBS-SSS=** Irritable Bowel Disease-Severity Scoring System;
21. **IL** = interleukin;
22. **ISI** = insulin sensitivity index;
23. **KYN** = Kynurenine
24. **LBP** = Lipopolysaccharide Binding Protein
25. **LDL** = low density lipoproteins;
26. **LPS** = lipopolysaccharide,
27. **MDS-UPDRS** (Movement Disorder Society-Unified Parkinson's Disease Rating Scale) = Parkinson's disease rating scale.
28. **Mice db/db** = mice homozygous for a defect in the leptin receptor;
29. **MMSE** (Mini mental State Examination) = cognitive status assessment tool;
30. **QOL** = Sleep quality and quality of life
31. **SCFA** = Short Chain Fatty Acids;
32. **scFOS** = short chain Fructo-oligosaccharide
33. **SNAP-IV** = ADHD assessment test;
34. **SNC** = Sympathetic nervous system;

35. **TNF** = Tumor Necrosis Factor;
36. **TRP** = Tryptophan;
37. **UC** = Ulcerative colitis.
